# Modulation of Gut Barrier Functions in Ulcerative Colitis by Hyaluronic Acid System

**DOI:** 10.1002/advs.202103189

**Published:** 2021-11-10

**Authors:** Niranjan G. Kotla, Isma Liza Mohd Isa, Swetha Rasala, Secil Demir, Rajbir Singh, Becca V. Baby, Samantha K. Swamy, Peter Dockery, Venkatakrishna R. Jala, Yury Rochev, Abhay Pandit

**Affiliations:** ^1^ CÚRAM, SFI Research Centre for Medical Devices National University of Ireland Galway Galway H91 W2TY Ireland; ^2^ Department of Microbiology and Immunology James Graham Brown Cancer Center University of Louisville Louisville KY 40202 USA; ^3^ Department of Anatomy National University of Ireland Galway H91 TK33 Ireland; ^4^ Present address: Department of Anatomy Faculty of Medicine Universiti Kebangsaan Malaysia

**Keywords:** dextran sodium sulfate (DSS)‐induced, HA, HA systems, inflammatory diseases

## Abstract

The active stages of intestinal inflammation and the pathogenesis of ulcerative colitis are associated with superficial mucosal damage and intermittent wounding that leads to epithelial barrier defects and increased permeability. The standard therapeutic interventions for colitis have focused mainly on maintaining the remission levels of the disease. Nonetheless, such treatment strategies (using anti‐inflammatory, immunomodulatory agents) do not address colitis' root cause, especially the mucosal damage and dysregulated intestinal barrier functions. Restoration of barrier functionality by mucosal healing or physical barrier protecting strategies shall be considered as an initial event in the disease suppression and progression. Herein, a biphasic hyaluronan (HA) enema suspension, naïve‐HA systems that protect the dysregulated gut epithelium by decreasing the inflammation, permeability, and helping in maintaining the epithelial barrier integrity in the dextran sodium sulfate‐induced colitis mice model is reported. Furthermore, HA‐based system modulates intestinal epithelial junctional proteins and regulatory signaling pathways, resulting in attenuation of inflammation and mucosal protection. The results suggest that HA‐based system can be delivered as an enema to act as a barrier protecting system for managing distal colonic inflammatory diseases, including colitis.

## Graphical abstract

Restoration of gut barrier function by mucosal healing or physical barrier protecting strategies shall be considered as an initial event in the colitis suppression and progression. The biphasic hyaluronan (HA)‐enema suspension and naïve‐HA treatments alter the dysregulated colon epithelium in dextran sodium sulfate‐colitis mice by reducing inflammation and permeability and improving gut barrier functions.

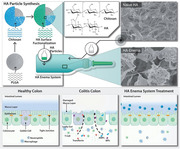

## 1 Introduction

Inflammatory bowel disease (IBD) pathogenesis is associated with gut mucosal inflammation,^[^ ^1^, ^2^ ^]^ epithelial dysregulation, and intestinal microbiome imbalance^[^ ^3^, ^4^ ^]^ resulting in gut barrier dysfunction.^[^ ^5^, ^6^ ^]^ The active stages of intestinal inflammation (in Crohn's and colitis) are linked to decreased transepithelial resistance and increased permeability.^[^ ^7^, ^8^, ^9^ ^]^ The elevated levels of reactive oxygen species (ROS) and metalloproteinases cause tissue degradation and necrosis induction.^[^ ^10^, ^11^ ^]^ Moreover, the inflammatory mediator elements are critical causative factors to mucosal inflammation and enhance overall permeability at the gut wall's ulcerative sites.^[^ ^12^, ^13^, ^14^ ^]^ Additionally, patients with IBD have decreased levels of tight junctional proteins (claudins, occludins) and junctional adhesion molecules (cadherins, catenins), leading to increased intestinal permeability resulting in systemic inflammation extraintestinal manifestations.^[^ ^7^, ^8^ ^]^

The standard therapeutic interventions for IBD include anti‐inflammatory, immunosuppressants, and biological medications, which have focused mainly on treating IBD symptoms by reducing inflammation, hyper immunity, and extraintestinal immune reactions.^[^ ^15^, ^16^, ^17^ ^]^ Patients with failure to aminosalicylate based drugs are treated with immunosuppressants, calcineurin inhibitors, which have generally been associated with off‐targeted systemic side effects (such as opportunistic infections, autoimmunity, hepatic toxicity, nephrotoxicity, and malignancies).^[^ ^15^, ^16^, ^17^ ^]^ In the second line of management, therapeutic agents such as corticosteroids are essential for inhibiting pro‐inflammatory cytokines; nonetheless, there is no evidence of intestinal epithelial restitution with the current medication systems.^[^ ^18^, ^19^, ^20^ ^]^ More importantly, existing drug therapies’ side effects cause substantial morbidity in patients, linked to immune system suppression brought about by current steroids and monoclonal antibody medications.^[^ ^21^, ^22^ ^]^ One such example is the use of monoclonal antibody agent infliximab to induce severe immune suppression and can result in severe and even life‐threatening infections.^[^ ^23^, ^24^, ^25^, ^26^ ^]^ Nonetheless, such treatment strategies do not generally address the root causes of various forms of IBDs.^[^ ^27^ ^]^

Restoration of gut barrier functions by mucosal healing (a physical barrier protecting strategy) can be an approach considered in the management of colitis.^[^ ^27^, ^28^, ^29^ ^]^ Intestinal mucosal healing, epithelial restitution, and symptom management have become essential to sustain the remission levels in various forms of IBDs. Although attempts have been made to bring new therapeutic agents, treatments for the mucosal repair, and tissue homeostasis in IBD are still under investigation.^[^ ^27^, ^30^ ^]^ Hyaluronan (HA) is one of the abundant components of the mucosal, epithelial, and extracellular matrix (ECM) of the intestinal wall of the gastric tract.^[^ ^31^ ^]^ These glycosaminoglycans (GAGs) polymeric networks are located below the epithelial barrier, ECM of the gut wall.^[^ ^32^, ^33^ ^]^ In addition to the anti‐inflammatory,^[^ ^31^, ^34^, ^35^ ^]^ microbiota enrichment properties^[^ ^36^ ^]^ of HA in intestinal inflammatory disorders, a few research reports also revealed that HA plays a crucial role in decreasing the intestinal permeability.^[^ ^31^, ^36^, ^37^ ^]^

Engineering one of the intestinal epithelial extracellular GAG, primarily naïve‐HA and HA‐based biphasic enema suspension system (as an enema dosage form administered into the lower GIT by the rectal route), forms a thick layer of dysregulated colitis areas of the gut and improves the gut barrier integrity and associated functions. Here, we report an HA‐enema suspension system and compared with naïve‐HA that can be administered as an enema (rectal dosage form), target to the inflamed colonic epithelium, provide a thick cementing protecting barrier layer, promote mucosal repair, and decrease permeability and enhance the overall integrity of the gut barrier functions.

## 2 Results

### 2.1 Design, Synthesis, and Characterization of HA‐Enema Suspension

**Figure 1:**
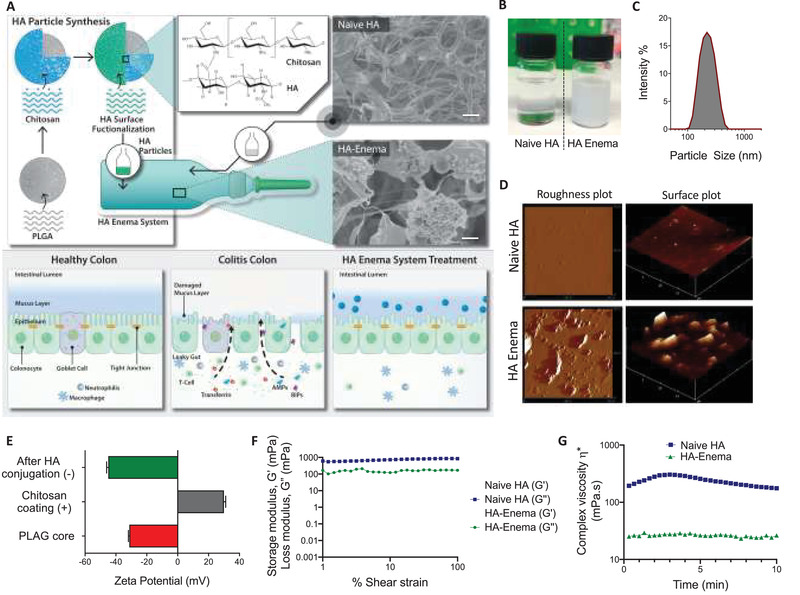
The HA‐enema suspension system's schematic and the proposed biophysical protective mechanism on the colitis epithelium, physicochemical properties. A) HA‐enema suspension system containing HA‐functionalized anionic polymeric nanoparticles dispersed in naïve HA solution, which can be easily administered as an intrarectal enema dosage form. A healthy colon epithelium has a thick mucosa layer and epithelial cells inlined with tight junctions to provide a cementing barrier to differentiate lumen contents from the systemic route. However, the dysregulated (colitis) colon wall has a damaged mucosal layer with loss of tight junction proteins, which creates a leaky gut epithelium. The HA‐enema system consisting of HMW‐HA solution will provide a physical protecting cementing barrier on the surface, HA particles will interact and penetrate at the colitis epithelium layers and may help in enhancing the barrier functions. SEM images of naïve HA and HA‐enema (scale bar = 1 µm). B,C) Representative formulation images of naïve HA, HA‐enema, and the particle size intensity % graph. D) Particle roughness and surface plots of AFM representative images. E) Change in the particle surface charge after each layer of coating. F) In both naïve‐HA and HA‐enema systems (3 mg mL^−1^), loss/viscous modulus (*G*″) is higher than storage/elastic modulus (*G*′), which indicates that both systems are viscous. G) Comparison of complex viscosity (mPa s) of naïve HA and HA‐enema over time.

We formulated the HA‐enema suspension system using high molecular weight sodium hyaluronate (1.2 × 10^6^ Da), consisting of HA‐functionalized polymeric nanoparticles dispersed in a naïve‐HA solution. The proposed mechanism of the device illustration was shown in **Figure** **1**A. Here, the enema suspension solution will provide a thick adhesive bio‐physical barrier on the epithelial surface, while the HA functionalized particles will preferentially accumulate in the inflamed colonic epithelium and interact/penetrate with epithelial cells. A representative scanning electron microscopy (SEM) image of the naïve HA and HA‐enema system is shown in Figure 1A, the Zetasizer was used to analyze the dispersed particle size in a HA solution system. The synthesized HA particles were mono‐dispersed, with the mean size range of 200–300 nm (Figure 1C), representative naïve HA (transparent solution), HA‐enema (free‐flowing, milky) formulations shown in Figure 1B. Atomic force microscopy (AFM) images showed increased roughness in the particle dispersed enema suspension system compared to the naïve HA solution. The mean roughness values of 3 mg mL^−1^ naïve HA, HA‐enema (particle to solution ratio: 1:2) were 26.6 and 269.8 nm, respectively. The naïve HA sample morphology offers a smooth and gel‐like appearance at the surface. The HA‐enema sample showed the nanoparticles spherically entangled in morphology in the HA‐solution's gel‐like nature (Figure 1D). These morphological features have been reported to influence cell adhesion and growth.^[^ ^38^, ^39^ ^]^

At each layer of the particle coating, the surface charge was changed from −31.6 mV (PLGA core) to +30 mV (after chitosan coating) and −45 mV after HA conjugation (Figure 1E). Understanding the viscoelastic behavior is critical as the HA‐enema system is administered directly into the rectal regions via the enema instillation procedure. Viscoelastic behavior of HA concentrations (3 and 6 mg mL^−1^) with different particle to solution ratios (1:2 and 1:4) was measured to determine the linear viscoelastic properties (modulus and viscosity) of naïve HA and HA‐enema systems. Particle to solution ratio did not affect the modulus and viscosity; all the formulations showed less viscosity with less than 1000 mPa s (fluid‐like system) (Figure S1, Supporting Information). The linearity of the modulus *G*′ and *G*″ under 0.1%–100% strain suggested the HA‐enema system's stability under strain behavior. For both naïve HA and HA‐enema of a representative concentration (3 mg mL^−1^), the *G*″ was higher than the *G*′ specifying that the HA system is viscous rather than elastic in behavior (Figure 1F). The maximum value for the viscosity of 257 mPa s was recorded for naïve HA and 26 mPa s for HA‐enema at 3 mg mL^−1^ concentration, which indicates that the particle form of the HA enema suspension system has a lower viscosity than that of the naïve HA (Figure 1G). The decrease in the viscosity of HA, in the enema form might be because of the shear thinning (pseudoplastic) fluids, at the applied shear rate the particles in the enema system are in the stream direction and lead to a decrease in the viscosity. In addition, the long‐chain biopolymer molecules in solution have a very open structure, especially hyaluronic acid, fill a large volume, leading to high viscosity. The addition of nanoparticles might stick to the polymeric networks; hence, surface structures are less open with lower volume, leading to less viscous. However, the observed difference is very minimal (both forms naïve HA and HA‐enema viscosities are in mPa s range).

### 2.2 Effect of HA‐Enema Suspension on Cellular Cytotoxicity, Inflammation, Permeability, In Vitro

**Figure 2:**
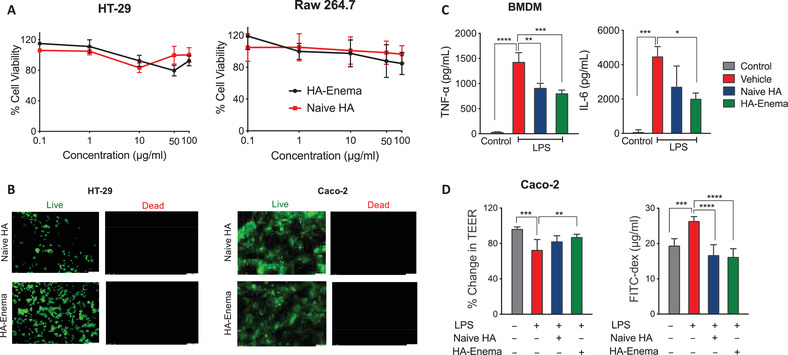
Naïve HA and HA‐enema effects on cells, in vitro. A) Cellular viability by MTT assay of naïve HA and HA‐enema (0.1, 1, 10, 50, and 100 µg mL^−1^) treatments. B) Representative Live/Dead images after naïve HA and HA‐enema (100 µg mL^−1^) treatment on HT‐29, Caco‐2 cell lines, scale bar 100 µm. C) BMDMs were stimulated with LPS (50 ng mL^−1^) with culture media alone (vehicle), naïve HA and HA‐enema (10 µg mL^−1^) treatments for 6 h, TNF‐*α* and IL‐6 levels in the culture supernatants. D) Monolayer Caco‐2 cells on transmembranes were treated with naïve HA and HA‐enema (50 µg mL^−1^) for 24 h, followed by treatment with LPS (50 ng mL^−1^) for 2 h. Change in % TEER and FITC‐dextran levels in the bottom chamber well was measured. The results were expressed for each treatment (*n* = 3); data were expressed as the mean ± SD. *P*‐values were determined by one‐way ANOVA with Tukey's post hoc test; ****p < 0.0001, ***p < 0.001, **p < 0.01, *p < 0.05 for LPS alone versus control and treatments.

The colon epithelial cells (Caco‐2 and HT‐29) and Raw 264.7 macrophages were used and examined by MTT assay and Live/Dead imaging analysis to evaluate the cellular cytotoxicity. MTT assay showed no cytotoxic effect of naïve HA and HA‐enema at various concentrations (0.1, 1, 10, 50, and 100 µg mL^−1^) (**Figure** **2**A). Additionally, no dead cell fluorescent signal was observed on HT‐29 and Caco‐2 cells with the highest concentration (100 µg mL^−1^) between the treatment groups with Live/Dead images (Figure 2B), indicating that HA‐enema is a suitable system that can be administered rectally as an enema dosage form to the colitis colon.

The bone marrow‐derived macrophages (BMDMs) were isolated from mice bone marrow and cultured using an existing published protocol.^[^ ^30^ ^]^ BMDM cells were used to test the naïve HA and HA‐enema effects on inflammation. The inhibition of LPS‐induced cytokine secretion (TNF‐*α*, IL‐6) by BMDMs was evaluated by comparing naïve HA and HA‐enema (10 µg mL^−1^). The HA‐enema was shown to decrease significantly (twofold) the pro‐inflammatory cytokines (measured by ELISA) when compared with the LPS alone (Figure 2C). The monolayer cell permeability was examined using the FITC‐dextran flux in a Transwell plate in vitro assay. As shown in Figure 2D, pre‐treatment of Caco‐2 cells with naïve HA and HA‐enema (50 µg mL^−1^) notably decreased the LPS‐induced FITC‐dextran flux. Intestinal epithelial junctional complex proteins maintain gut barrier integrity and permeability; these proteins’ levels are significantly downregulated under inflamed conditions, leading to increased intestinal permeability.^[^ ^7^, ^40^ ^]^ Moreover, treatment with naïve HA and HA‐enema upregulated expression of the tight junction proteins claudin4 (Cldn4) and occludin (Ocldn) in Caco‐2 cells (Figure S2, Supporting Information).

### 2.3 HA‐Enema Suspension Ameliorates DSS‐Induced Acute Colitis in Mice

**Figure 3:**
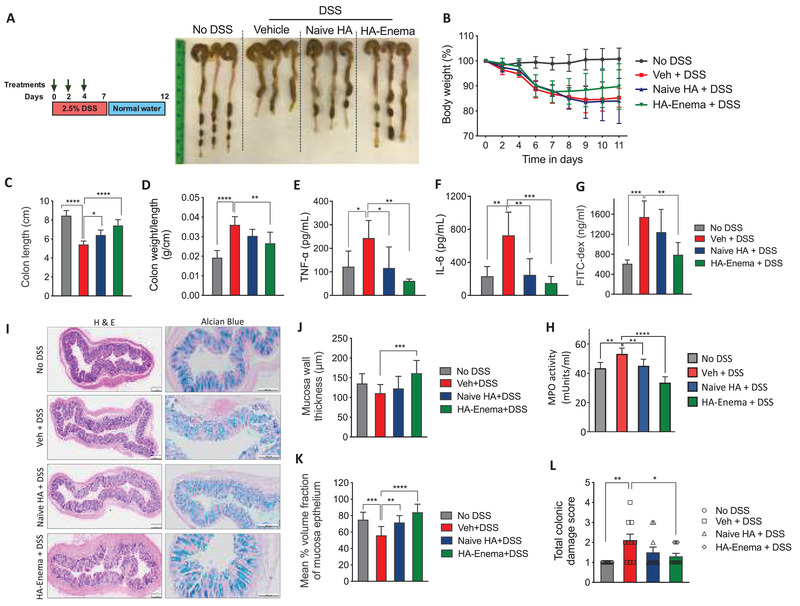
HA‐enema suspension therapeutic efficacy in DSS colitis mice model. A) C57BL/6 mice (age: 6–8 weeks) were treated with DSS (2.5%) in drinking water for 7 d, followed by 5 d with regular water. The control group of mice received regular water without DSS. Mice were rectally administered with vehicle (PBS) or naïve HA and HA‐enema (30 mg kg^−1^ body weight) on day 0, 2, 4. Mice were euthanized on day 12, and the colitis phenotype was assessed, the representative gross morphological changes of colon images. B–D) The percentage of body weight loss, colon lengths, and the ratio of colon weight/length. E–G) Serum levels of TNF‐*α* and IL‐6, FITC‐Dex flux‐permeability. Statistical analysis was performed using GraphPad Prism software by one‐way ANOVA with Tukey's post hoc test. Data were expressed as the mean ± SD. ****p < 0.0001; ***p < 0.001, **p < 0.01; *p < 0.05. H) MPO activity (milliunits mL^−1^) were determined in colon tissue homogenate solutions. I–L) Images of H&E, Alcian blue‐stained mucosa sections of colons, stereological estimation of total mucosal wall thickness, mean % volume fraction of mucosa epithelium, and total colitis score. Scale bar indicates 200 µm. Statistical analysis was performed using GraphPad Prism software by one‐way ANOVA with Dunnett's multiple comparisons test. Data were expressed as the mean ± SEM. ****p < 0.0001; ***p < 0.001; **p < 0.01; *p < 0.05.

We also investigated the naïve HA and HA‐enema therapeutic efficacy in a DSS‐induced acute colitis mice model. Rectal administration with naïve HA and HA‐enema (30 mg kg^−1^ at day 0, 2, and 4) was considerably protected from DSS‐induced body weight loss. Analysis of harvested colons suggested that HA‐enema treatment protected from DSS‐induced colon shortening and reduced weight to length ratio, indicating decreased colonic inflammation (**Figure** **3**A–D). Further, naïve‐HA and HA‐enema treatment reduced serum inflammatory markers (IL‐6, TNF‐*α*) compared to vehicle in DSS‐induced colitis mice. Importantly, HA‐enema treatment significantly reduced DSS‐induced intestinal permeability, as evident from FITC‐dextran leakage into serum upon oral delivery (Figure 3E–G) and myeloperoxidase enzyme (MPO) levels (Figure 3H). Consistent with these findings, hematoxylin and eosin (H&E) and mucosa stained (Alcian blue) images (Figure 3I) of colon tissue sections demonstrated considerably less tissue damage and histological inflammation by stereological assessments of mucosa thickness, % volume fraction of colonic mucosa epithelium (Figure 3J,K). The total colitis damage score (Figure 3L) was determined by blinded histopathological analysis, by adapting the scoring parameters as reported in the literature (inflammatory cell infiltration on the mucosa, submucosa level score of 0–3; intestinal architecture with focal erosions, extended ulcerations score of 0–3; the total score was given on a scale of 0–6).^[^ ^30^, ^41^ ^]^ The H&E stereological and histopathological analysis showed that the HA systems protected against the DSS‐induced colitis and showed minor tissue damage after the treatment.

### 2.4 Proteomic Changes Underlying HA‐Enema Suspension Treatment

**Figure 4:**
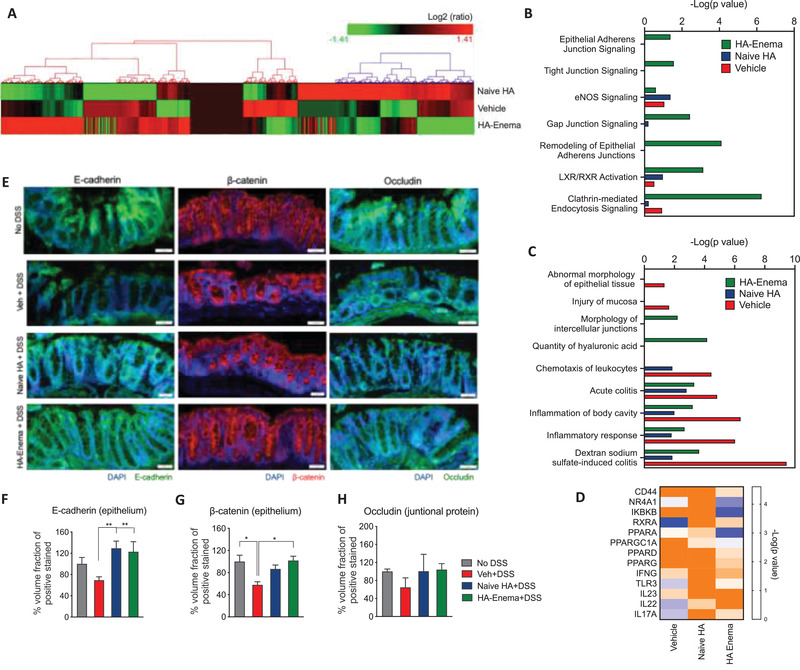
Major proteomic changes in response to HA‐enema suspension treatment in DSS colitis mice model. A) Differentially expressed proteins were shown in heatmaps of log2‐transformed abundance generated by Hierarchical Clustering Explorer 3.0, with red for high expression and green for low expression. B) The quantified activity score of canonical pathways for the vehicle and naïve HA, HA‐enema treatments. C) The quantified activity score of significant cellular/molecular disease pathological pathways for the vehicle and naïve HA, HA‐enema treatment as determined by IPA. D) Heatmap of the top putative upstream regulators increasing (blue) and/or decreasing (orange) involved in modulated cellular functions with a −log 10 *p*‐value of 1.0 in DSS colitis model and the vehicle (PBS) and naïve HA, HA‐enema treatments. E) Representative immunofluorescence images showing increased E‐cadherin (epithelium), *β*‐catenin (epithelium), occludin (adherent junctional proteins) at the colitis epithelium site with naïve HA and HA‐enema group compared to vehicle (PBS) control group and F–H) respective quantifications. Statistical analysis was performed using GraphPad Prism software by one‐way ANOVA with Dunnett's multiple comparisons test. Data were expressed as the mean ± SEM. **p < 0.01; *p < 0.05.

Protein expression of the vehicle (PBS), naïve HA, and HA‐enema treated DSS colitis colon tissue samples were analyzed by liquid chromatography coupled to tandem mass spectrometry (LC‐MS/MS). In total, 4228 common proteins (containing at least two unique peptides) between two tandem mass tag labeling (TMT) sets were successfully quantified (false discovery rate, FDR 1% and *p* < 0.01). To indicate the overall proteome profile, the hierarchical clustering was applied to protein data matrix (log(2) fold change >1 or ←1 for log2 expression values, *p*, 0.05), which the heatmaps show the proteins spread across clusters with differentially expression levels in the DSS vehicle group, DSS with naïve‐HA and HA‐enema treated groups (**Figure** **4**A). To identify the critical signaling pathways, a total of 578 differentially expressed proteins when compared to the control group (log (2) fold change cut off 1.0, *p* < 0.05) was analyzed by ingenuity pathway analysis (IPA). IPA was used to identify the predicted canonical pathways, associated upstream regulators and the biological functions significantly enriched (−log 10 *p*‐values 1.0) in a temporally specific manner post‐treatment with PBS (vehicle), naïve HA, and HA‐enema treatment in a DSS colitis mice model.

We found that the epithelial adherens junction signaling, tight junction signaling, remodeling of epithelial adherens junctions, gap junction signaling, clathrin‐mediated endocytosis signaling, nuclear receptor signaling via LXR/RXR (liver X receptor, LXR; retinoid X receptor, RXR) highly enriched by the differentially expressed proteins in the vehicle (PBS), naïve HA, HA‐enema treatment groups (Figure 4B) (−log 10 *p*‐values relative to control group). The altered proteins that identified the activation and inhibition of canonical signaling were associated with biological functions based on their target proteins’ expression. The HA particles in the suspension system shown to be uptaken by epithelial cells of the colitis intestinal wall by clathrin‐mediated endocytosis signaling (−log 10 *p*‐values of HA‐enema 6.3 compared to vehicle 0.9 and naïve HA 0.2) indicates that the HA in the particle form has more efficient interaction with epithelial cells than with HA in its naïve form (Figure 4B). The epithelial junctional signaling process (including tight junction, gap junction, adherent junctions) increased with HA‐enema treatments compared to that of the vehicle (PBS) and naïve HA treatment. Besides, the nuclear receptors also control the intestinal homeostasis, involve in nutrient intake, helps in the clearance of xenobiotics, toxic dietary components, and absorption/metabolism of bile acids or cholesterol by LXR / RXR pathways.^[^ ^42^, ^43^ ^]^ HA‐enema activated the LXR/RXR pathways compared to vehicle or naïve HA treatment (−log 10 *p*‐values of HA‐enema 3.1 compared to vehicle 0.5 and naïve HA 1.0) (Figure 4B).

The disease and biological response that reflects the colitis pathology includes the potent inhibition of mucosal inflammation, abnormal morphology of the epithelial tissue, intracellular junctional function, epithelial wall remodeling, and intestinal homeostasis. The proteins involved in the intestinal inflammation or colitis condition underwent a dramatic downregulation after HA‐enema treatment. As a result of this, specific inflammation response with dextran sodium sulfate‐induced colitis (−log *p*‐value of vehicle treatment: 9.4 to naïve HA (1.9), HA‐enema (3.6), acute colitis (−log *p*‐value of vehicle treatment: 4.8 to naïve HA (2.8), HA‐enema (3.3), inflammation of body cavity (−log *p*‐value of vehicle treatment: 6.4 to naïve HA (2.0), HA‐enema (3.2) is significantly reduced (Figure 4C). The top upstream regulators based on their target proteins’ expression were shown as a heatmap (Figure 4D) image. Among the upstream proteins identified as being differentially regulated with naïve HA and HA‐enema treatment compared to vehicle (DSS) treatment in the DSS colitis mice model, the epithelial adherent/tight junctional proteins (E‐cadherin, *β*‐catenin, occludin), and cell surface receptor of HA, CD44 were selected for protein validation. CD44 is a transmembrane glycoprotein, it is been observed that in many tumors and colitis tissues, an increased expression of CD44 has been observed where in HA binding and internalization are more significant, which plays a key role in HA internalization, degradation of ECM components, cell adhesion, proliferation, and migration.^[^ ^34^, ^44^, ^45^ ^]^ Representative immunofluorescence images showing increased E‐cadherin, *β*‐catenin (epithelium), occludin (adherent junctional proteins) at the colitis epithelium with naïve HA and HA‐enema group compared to vehicle (PBS) control group (Figure 4E–H). Similarly, the surface receptor (CD44) staining was also shown in Figure S3 (Supporting Information). Additionally, the regulated inflammatory markers that were inhibited in IPA analysis were further validated by ELISA with protein lysates of control, vehicle (PBS), naïve HA, HA‐enema treated colitis tissue samples by U‐plex biomarker cytokines panel (mice) for cytokines (IL‐1*β*, IL‐6) (Meso Scale Discovery) (Figure S4, Supporting Information).

## 3 Discussion

Ulcerative colitis is one of the primary forms of IBD and is associated with the colon mucosal pattern inflammation, epithelial dysregulation resulting in functional abnormalities of the overall gut barrier.^[^ ^46^ ^]^ Dysregulated colon epithelial barrier functionalities primes to leakage of the colon luminal contents flux into the systemic route, causing extraintestinal manifestations including uveitis, iritis, erythema, and arthritis.^[^ ^47^, ^48^ ^]^ The conventional anti‐inflammatory, immunosuppressant treatments do not resolve the underlying causes of IBDs, including colon damage to the mucus layer and subsequent loss of intestinal barrier functions. There is, thus, a pressing need for new, more effective symptom relief treatments (including gut wall healing, mucosal repairing, permeability decreasing agents) in bowel inflammation specific to colitis.

The current work reported here demonstrated the potential beneficial role of HA in the modulation of the dysregulated DSS‐induced colitis condition. The HA‐enema rectal delivery system was developed using the fabricated HA functionalized polymeric particles dispersed in the HA solution. The formulated HA bi‐phasic system has the potential future advantage of delivering drugs precisely by loading into the particles for a combinatorial therapy for distal colitis therapy. The concept of size‐dependent particles, surface‐functionalized carriers on intestinal mucoadhesion is well explored^[^ ^49^, ^50^ ^]^ with associated signaling mechanisms including endocytosis.^[^ ^51^, ^52^ ^]^ The therapeutic or beneficial effects depend on the size, biopolymer type (including hyaluronic acid). The smaller the particle size (nano) better the adhesion on the intestinal tract. The authors presume that surface functionalization of HA on the polymeric nanoparticle system (as an enema form) help in improving the barrier functions by interacting on the mucosal surface, particles endocytosis into the epithelium compared to naïve HA solution. The synthesized HA particles were monodispersed with a 200–300 nm size range, with an overall negative surface charge of −45 mV. AFM images showed increased roughness in the particle dispersed solution system compared to that in the naïve HA solution. We observed no cytotoxicity on colon epithelial macrophage cell lines with any given concentrations of naïve HA or HA‐enema by MTT assay and Live/Dead staining. One of our research's significant observations includes reducing TNF‐*α*, IL‐6 on BMDMs after treating HA treatments compared to the LPS‐treated group. Additionally, there was an increase in transepithelial electrical resistance (TEER) and a reduction of FITC‐dex flux across the Transwell membrane after treating with HA‐enema when compared to LPS alone. Further in vivo (DSS colitis mice model) study revealed that the HA‐enema system has a promising therapeutic potential in managing colitis and helps maintain the integrity of the gut barrier, and further likely to prevent the disease's progression from the colon lumen endotoxins.

**Figure 5:**
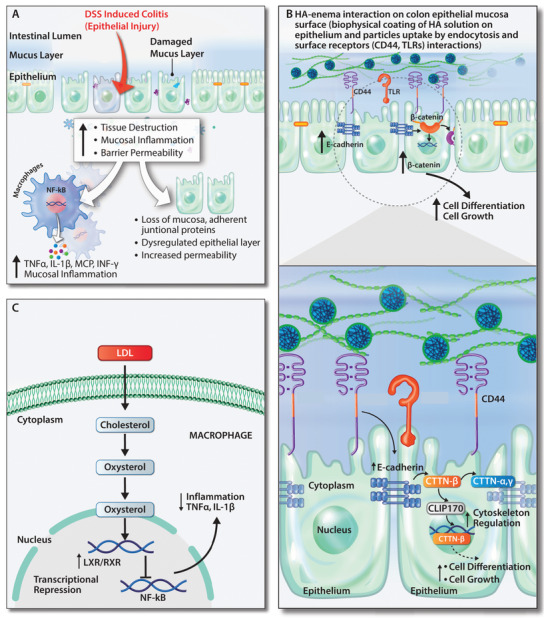
Possible mechanistic role of the HA‐enema suspension in the DSS induced colitis mucosal epithelium. A) External mucosal inflammation trigger (DSS solution) in the intestinal lumen causes the epithelium/mucosal damage. In downstream causes destruction of the tissue, inflammation of the colon epithelium, and an increase in the overall gut wall's permeability. B) Biphasic HA‐enema favorably provides mucoadhesive cementing protective barrier effect. The HA‐functionalized polymeric particles, HA solution both interact with cell surface receptors (including CD44, TLRs), upregulate the epithelial adherent junctional proteins E‐cadherin, *β*‐catenin which regulate the cytoskeleton, cell growth and repair on the dysregulated colitis wall. C) The LXR/RXR pathways were activated by HA‐enema via LDL uptake in macrophages and activate the LXR/RXR pathway, inhibiting the inflammation (NF‐kB) pathway leads to decreasing inflammation. The developed HA‐enema system serves as physical surface matrix barrier and protects from DSS external stimuli and might help in maintaining the gut barrier integrity in a DSS colitis mice model.

We were further motivated to find the possible role and mechanism of action of both forms of HA (naïve HA and HA‐enema suspension) in the DSS‐induced colitis mucosal epithelium (**Figure** **5**). We understand that the particles were uptaken by endocytosis and also due to the possibility of binding to surface receptors on the colitis inflammatory gut wall,^[^ ^53^ ^]^ upon interaction of HA system, in downstream, there was an upregulation of transcription of genes that encode adherent epithelial/junctional proteins including E‐cadherin, *β*‐catenin, occludin, and inhibition of pro‐inflammatory cytokines. Both forms of HA systems (majorly HA‐enema), possibly via epithelial adherent junctional signaling (by E‐cadherin), promotes cell growth regulation, cytoskeleton rearrangements. A higher expression of E‐cadherin, catenins (*α*, *β*, *γ*) in the intestinal epithelial cells and up‐regulation of adherent junctional proteins (such as occludins) mediate the enhancement of the gut barrier integrity and decreasing the permeability. Additionally, HA‐enema activates the LXR/RXR pathway, which further suppresses the inflammation mediated by the NF‐kB pathway in the mucosal inflammation of the acute colitis models. Further downstream regulatory molecular pathways need to be established to define the HA‐based protective activities in intestinal inflammation.

Our findings illustrate the efficacy and protection of HA‐enema in colitis and indicate that both HA‐based systems play an essential protecting role against DSS‐induced colitis. The current research demonstrates the rectal HA‐enema used as an amending mucoadhesive coat on the damaged colon barrier, modulating intestinal barrier inflammation and permeability. However, testing the system effect in a single acute colitis model, unavailability of rectal region‐site‐specific colitis mice models are the key limitation of the study. In addition, testing in chronic and large animal models, investigating further on the possible mechanistic aspects (including receptor level mechanistic effects, understanding the barrier protective/epithelial repair processes, effect on the colon microbiome) are essential. Besides, the potential benefits with the current HA‐enema system are the possibility to encapsulate any model therapeutic drugs (especially corticosteroids, immunosuppressants, or disease modifying drugs) in the particles, which may help in effective combinatorial therapies in distal colitis. Due to the close association of dysregulated intestinal barrier functions with systemic diseases, the current biophysical protecting barrier strategy with HA‐enema holds promise and may help in restoring the dysregulated intestinal barrier functions and help in the distal colitis management.

## 4 Experimental Section

### Materials

Sodium hyaluronate (Mw 1.2 × 10^6^ Da) was purchased from Lifecore Biomedical, USA. Polyvinyl alcohol (Mw 13k–23k Da), 4‐(4.6‐dimethoxy‐1,3,5‐triazin‐2‐yl)‐4‐methylmorpholinium chloride (DMTMM), PLGA (Resomer RG 504H, poly(d,l‐lactide‐*co*‐glycolide); 50:50, average Mw ≈ 38k–54k Da), and chitosan (average Mw ≈ 50k–190k Da) were purchased from Sigma‐Aldrich. LPS, FITC‐dextran (Mw 4k Da), Alcian blue, Nuclear Fast Red were purchased from Sigma‐Aldrich. ELISA kits for IL‐6 and TNF‐*α* were purchased from Bio‐legend, Multiplex ELISA pro‐inflammatory panel was purchased from Meso Scale Discovery. E‐Cadherin monoclonal antibody (Alexa Fluor 488 conjugate; eBioscience, Thermo Scientific), recombinant anti‐beta catenin antibody (Alexa Fluor 568 conjugate; ab201823), Human/Mouse CD44 antibody (Alexa Fluor 488 conjugate; R&D systems), occludin, mouse IgG1 Alexa Fluor 488, *β*‐actin from Santa Cruz Biotechnology, and DAPI purchased from Life Technologies. Colitis grade DSS (Mw 36k–50k Da) was from MP Bio, and other reagents used were of analytical grade.

### Synthesis and Fabrication of HA‐Enema Suspension System

The hyaluronic acid suspension system was fabricated using high molecular weight sodium hyaluronate (1.2 × 10^6^ Da). The enema suspension system consists of HA‐functionalized PLGA core nanoparticles, which were dispersed in HA solutions of different concentrations. The HA‐functionalized polymeric nanoparticles were synthesized as per a previous publication.^[^ ^54^ ^]^ HA‐functionalized particles were dispersed in HA solution in different final concentrations (3, 6 mg mL^−1^) with a 1:2 ratio of HA particle to HA solution to prepare HA‐enema system. The naïve HA solutions were prepared in PBS using a commercially available source (Lifecore Biomedical).

### Physicochemical and Morphological Characterization

The particle size and the surface charge of HA particles in the suspension were analyzed by Malvern Zetasizer. The morphology of the nanoparticles was analyzed by SEM. Besides, the HA‐enema suspension system's surface roughness was compared to naïve HA by AFM. Rheological measurements of naïve HA and HA‐enema systems were performed with the Anton Paar Modular Compact Rheometer (MCR) 302 using a parallel plate (25 mm). In the preliminary experiments, the measurement parameters were determined by amplitude and time sweeps within the linear viscoelastic region. Amplitude sweep (1–100% shear strain) was used to measure the deformation behavior at a constant frequency (1 Hz) and 37 °C. Besides, HA‐enema viscoelastic changes were measured with time sweep (10 min) by maintaining frequency (1 Hz) and amplitude (10% shear strain) constant. Rheological measurements were recorded as storage modulus (*G*′), loss modulus (*G*″), and viscosity (n*), respectively.

### Cell Culture

Human colon epithelial carcinoma cell lines, HT29 and Caco2 cells, and Raw 264.7 macrophage cell lines were used to study cytotoxicity and permeability of the naïve HA‐enema. MTT assay for assessing cell viability: In brief, HT‐29 and Raw 264.7 were grown in a 96‐well tissue culture plate (10K per well) for 24 h. Naïve HA and HA‐enema systems were treated with concentrations (0.1–100 µg mL^−1^) for 24 h. After incubation, 10 µL of MTT reagent was added (final concentration 0.5 mg mL^−1^) and incubated for 3–4 h, at 37 °C. All the supernatants from the above wells were removed. A solubilizing agent (DMSO 100 µL) was added to each well and kept in the dark for 10 min, and absorbance was measured at 570 nm. The control cells absorbance was considered 100% and compared to the treatments. Live/dead imaging analysis was carried out to determine the effects of the naïve HA and HA‐enema system on HT‐29 and Caco‐2 cell viability as per a previous publication.^[^ ^54^ ^]^ Anti‐inflammatory effect on BMDMs: In a 96 well plate, 10 000 BMDM cells/well were plated for 24 h to adhere. The naïve HA and HA‐enema were treated with 10 µg mL^−1^ along with LPS for 6 h (LPS: 50 ng mL^−1^). After 6 h of incubation, supernatants were analyzed for cytokines by following the company provider's ELISA kit protocol (BioLegend). Cellular permeability studies: Briefly, colon epithelial‐like cancer cells (Caco‐2) were seeded on membrane filters (5 × 10^4^ per well) in 24‐well Transwell plates (Corning, USA) as per the published protocol,^[^ ^30^, ^55^ ^]^ quantity sufficient culture medium was added to the apical, basolateral chambers, media was changed every alternate day for 21 d. The TEER was measured using an EMD Millipore Millicell‐ERS2 Volt‐Ohm Meter (Millipore) during the process. Cell inserts with >1000 Ω cm^2^ were used further for permeability assessment. Cells were pretreated with naïve HA, HA‐enema (50 µg mL^−1^) for 24 h, respectively, and washed with PBS (3×). To each well, LPS solution (50 ng mL^−1^) was added (200 µL) and incubated for 2 h and washed with PBS (2×). Further, 200 µL of FITC‐Dex solution (1 mg mL^−1^ in HBSS) was added in the apical/upper chamber for 2 h, and the flux (FITC‐Dex) was determined using a plate reader at 480 and 525 nm, respectively. Besides, to analyze tight junctional proteins (claudin and occludin), total protein lysates were obtained from cells (Caco‐2) using the RIPA (Sigma‐Aldrich, USA) buffer and quantified by BCA (Thermo Scientific) protein kit as per manual instructions. The western blot protocol was adapted from a previous publication.^[^ ^30^ ^]^

### Mice

C57BL/6 mice were purchased from Jackson Laboratories, USA and Charles River, UK Laboratories. Mice that were between 6 and 8 weeks of age for all the experiments were used. Animals were kept in specific pathogen‐free (SPF) barrier environments, with dark and light cycles, allowed access to food and water. All the experiments were conducted under authorized protocols from the Institutional Animal Care and Use Committee (IACUC), University of Louisville, Louisville, KY; and Animal Care Research Ethics Committee at the National University of Ireland, Galway, and the Health Product Regulatory Authority, Ireland.

### DSS‐Induced Colitis

Acute colitis in mice (C57BL/6, 6–8 weeks old, *n* = 5–6 per group, repeated the same number of animals in a similar independent study) was induced by giving 2.5% (w/v) DSS (Mw ≈ 36 000–50 000 colitis grade, MP Biomedicals) in drinking water for 7 d and with a recovery period in regular drinking water for 5 d. Control animals received drinking water without DSS. Naïve HA and HA‐enema (30 mg kg^−1^ body weight) dose were given for respective DSS groups rectally by gavage tube on day 0–4 (under anesthesia). The animals were regularly observed for % body weight loss and behavior during the study. On day 12, all the mice were euthanized, underwent tissue harvesting, plasma collection, and examined for colitis phenotype markers.

### Assessment of Colitis Severity and Tissue Collection

During the study, all mice were regularly observed/given a score (on a scale of 9) based on % body weight changes, stool consistency, and rectal bleeding, combined to obtain the disease activity index (DAI) score.^[^ ^30^ ^]^ After euthanasia, blood was obtained from the abdominal vein and centrifuged (3500×*g*) for 15 min to obtain serum for cytokine analysis. Each colon was separated and cleaned with PBS; the colon length and weight were recorded. Each colon was cut into pieces and preserved for histological examination, protein and other colitis phenotype analysis.

### In Vivo Intestinal Permeability, Inflammation, and MPO Levels Assessment

The in vivo intestinal permeability in mice was analyzed using FITC‐dextran (Mw 4000) flux from the colon wall to the systemic route as per a previous publication.^[^ ^30^ ^]^ Briefly, FITC‐dextran (60 mg/100 g body weight) was given to mice (on fasted state: 4 h) orally, and the FITC‐dextran levels in serum were determined at Ex 485 nm and Em 525 nm, respectively, by plate reader. The collected blood from the abdominal vein was centrifuged at 3500×*g* for 15 min to separate serum. For cytokine assessment via ELISA, cytokines (IL‐6 and TNF‐*α*) from the collected serum were quantified by specific ELISA kits (Biolegend) as per the kit instructions. For colonic MPO activity a colorimetric assay kit was used (MAK068, Sigma‐Aldrich). The MPO activity in milliunits mL^−1^ was determined as per the kit instructions.

### Histopathology and Stereology Quantification

H&E staining: DSS colitis study tissues fixed in 10% formaldehyde solution underwent tissue processing before paraffin embedding. The paraffin sections of 5 µm were cut (using a Leica microtome), stained for H&E, and dried overnight. The H&E images were taken with a light microscopy unit/slide scanner. The stereological assessment analysis was adopted to determine the mucosa wall thickness and % volume fraction of colonic mucosa epithelium. Histological staining of mucins: Alcian blue stains acid mucosubstances and acetic mucins such as GAGs. The sections were deparaffinized and hydrated using standard lab protocol. Sections were then stained with Alcian blue (pH 2.5) (Sigma B8438, 1% in 3% acetic acid) for 20 min (which stains acidic mucins a light blue) and rinsed in tap water. Sections were counterstained for 5 min with nuclear fast red and rinsed in running tap water, dehydrated in 100%, 70%, and 50% ethanol 2 min each and cleared in xylene for 20 min, and mounted with a coverslip, dried overnight and imaged.

### Global Quantitative Proteomic Analysis by LC‐MS

To determine the total proteome of healthy/control, disease (DSS+vehicle), treatment 1 (DSS+ naïve HA) and treatment 2 (DSS+ HA‐enema) mouse colon tissues, global quantitative proteomics analysis was performed at DC Biosciences as per their protocol. In each group, three tissues underwent the proteomic analysis. The mouse colon tissues were lysed in bead beater tubes with 100 × 10^−3^ m Tris pH 8.5, sodium deoxycholate (SDC) 1%, 10 × 10^−3^ m TCEP, and 40 × 10^−3^ m chloroacetamide supplemented with a Roche protease inhibitor cocktail. The lysis was carried out by bead beating at max speed 4 × 20 s with 1 min intervals on ice. Samples were boiled for 5 min, and after centrifugation (20 000×*g*, 10 min), the supernatant was used to evaluate further. Total protein was quantified using the fluorescence‐based EZQ assay (Life Technologies). For protein digestion, trypsin was used (enzyme to substrate ratio of 1:50 (w/w). The digestions were carried out at 37 °C for 18 h and stopped by formic acid (FA) acidification to 2% (v:v) final concentration. Acid precipitation (pH 2.0) with 2% (v/v) FA was obtained by removing SDC after a trypsin digest from the cell lysates with 1% SDC. The peptides were desalted and dried using C18 Sep‐Pak cartridges as instructed by the manufacturer. The peptide concentration was determined using the Thermo Scientific Pierce Quantitative Fluorimetric Peptide Assay. Peptides were reconstituted in 100 × 10^−3^ m (TEAB, triethyl ammonium bicarbonate), and TMT labeling was carried out on 80 µg of peptides from each sample. Samples were randomly distributed in the 9‐plex label set. Mass spectrometry data acquisition was carried out by high pH reversed‐phase chromatography and nano‐LC mass spectrometry. The mean abundances ratios between the three given groups (healthy, diseased, treated) were calculated.

### Protein Quantification and Pathway Analysis

IPA (QIAGEN) was used for comprehensive proportional bioinformatics analysis of differentially expressed proteins (DEPs) in response to DSS‐induced colitis after treatments with naïve HA and HA‐enema. A suite of IPA algorithms and methods were used to classify significantly altered canonical pathways, downstream biological functions and upstream regulators. A log2 fold change cut‐off was kept −1.0 to 1.0, and the *p*‐value cut‐off criteria for the enrichment was −log 10 (*p*‐value) > 1.3. For the clustering analysis, data were normalized to healthy control, and the clustering analysis was conducted using Hierarchical Clustering Explorer 3.0 (NIH) without additional normalization. The results are shown in the heatmap format. The protein expression comparison between the disease (acute DSS colitis) and naïve HA, HA‐enema groups of colon tissues was analyzed with an empirical test based on fold change value as counts, causal networks (such as acute colitis, ulcerative colitis, mucosal injury, ECM, epithelium morphology, intestinal inflammation), and species accordingly.

### Assessment of Proteomic Changes Underlying Intestinal Epithelium

Bioinformatics analysis has shown that unique protein subsets significantly altered in each experimental paradigm. Among the proteins identified as differentially regulated, the epithelial adherent and junctional proteins (E‐cadherin, *β*‐catenin, occludin) and CD44 staining were selected for biological validation. In brief, the colon sections underwent deparaffinization using xylene and were subsequently rehydrated in ethanol (different percentages). Antigen retrieval was executed with an in‐house high‐pressure cooking system in 10 × 10^−3^ m Tris/1 × 10^−3^ m EDTA buffer at pH 9.0 for 18 min. The sections were washed in dH_2_O for 5 min (3×) and incubated in 3% hydrogen peroxide for 10 min, followed by (3×) washings in dH_2_O for 5 min. To block nonspecific staining, the slides were incubated with 5% normal goat serum in tris buffered Saline with 0.1% Tween‐20 (TBST) for 1 h at room temperature. After removing the blocking solution, primary antibody incubation was performed overnight at 4 °C. All the antibodies E‐cadherin monoclonal antibody (Alexa Fluor 488, conjugate), recombinant anti‐beta catenin antibody (Alexa Fluor 568 conjugate), occludin antibody (E‐5), and Human/Mouse CD44 antibody (Alexa Fluor 488‐conjugate) were used at a dilution of 1:100. Secondary antibody incubation was performed for Occludin staining with mouse IgG1 Alexa Fluor 488 for 1 h at room temperature, dark condition, and slides were washed (3×) afterward for 10 min in darkness. The coverslip was mounted by placing a drop of flour mount with DAPI. All slides were stored at 4 °C, dark environment before imaging with a slide scanner unit (Olympus, Digital Slide Scanner VS120FL).

### Statistical Analysis

Statistical analysis was performed using GraphPad Prism Version 8. Data were compared using one‐way analysis of variance (ANOVA) based on the number of factors analyzed by one‐way ANOVA with Tukey's post hoc test and Dunnett's multiple comparisons test. Statistical significance was set at *p < 0.05. All error bars indicate SD except SEM for proteomic validation markers analysis.

## Conflict of Interest

The authors declare no conflict of interest.

## Author Contributions

N.G.K. designed, performed, and analyzed all the experiments, surgeries, and wrote the manuscript. I.L.M.I., R.S., D.S., S.K.S., B.V.B., and S.R. performed and assisted with animal experiments. P.D. assisted with stereological analysis. V.R.J. designed, discussed, and analyzed all the in vitro and preclinical studies. Y.R. and A.P. directed the project and discussed all the results. All authors contributed to the manuscript preparation and approved the final manuscript.

## Supporting information

Supporting Information

## Data Availability

Research data are not shared.
