# Supplementary material for: Modulation of Gut Barrier Functions in Ulcerative Colitis by Hyaluronic Acid System
Source: Adv Sci (Weinh). 2021 Nov 10;9(4):2103189. doi: 10.1002/advs.202103189 (PMC8811821; doi:10.1002/advs.202103189)
Supplement: Supplementary file 1 — Supporting Information [file ADVS-9-2103189-s001.pdf]

© 2021 Wiley-VCH GmbH

ADVANCED SCIENCE
Open Access

Supporting Information

for *Adv. Sci.*, DOI: 10.1002/advs.202103189

Modulation of gut barrier functions in ulcerative colitis by hyaluronic acid system

Niranjan G. Kotla, Isma Liza Mohd Isa, Swetha Rasala, Secil Demir, Rajbir Singh, Becca V. Baby, Samantha K. Swamy, Peter Dockery, Venkatakrishna R. Jala, Yury Rochev, Abhay Pandit**

SUPPLEMENTARY INFORMATION

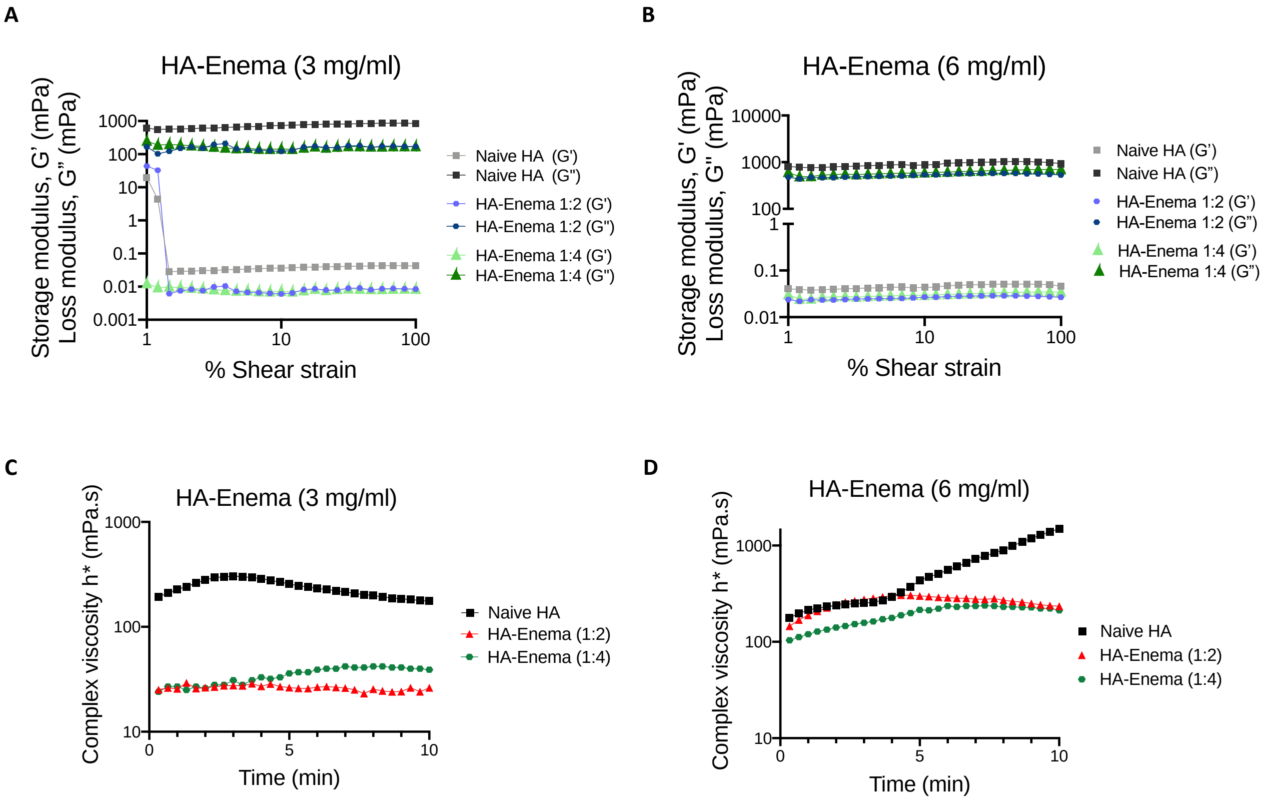

Figure. S1. Rheological measurements of Naïve HA and HA- enema suspension system.

(A, B) Storage modulus (G'), loss modulus (G'') and complex viscosity ($\eta *$) values for 3 mg/mL formulations of Naïve HA, HA-enema (1:2) and HA-suspension (1:4) solutions over a percent shear strain range of 1-100% and time sweep respectively. (C, D) Storage modulus (G'), loss modulus (G'') and complex viscosity ($\eta *$) values for 6 mg/mL formulations of Naïve HA, HA-enema (1:2) and HA-enema (1:4) solutions over a percent shear strain range of 1-100% and time sweep respectively.

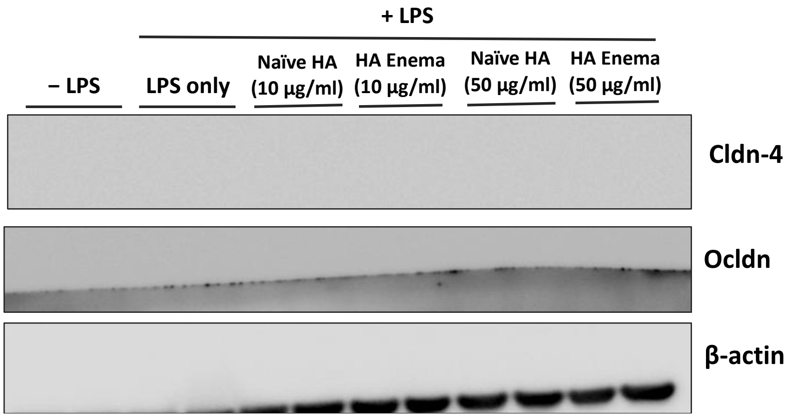

Figure S2. Effect of Naïve HA and HA-enema treatment on Caco-2 cells. Monolayer Caco-2 cells were treated with Naïve HA and HA-enema (10, 50 µg/mL) for 24 hrs followed by treatment with LPS (50 ng/ml) for two hrs. Naïve HA and HA-enema moderately induced protein expression of Cldn4, Ocln in Caco-2 cells was determined by immunoblots.

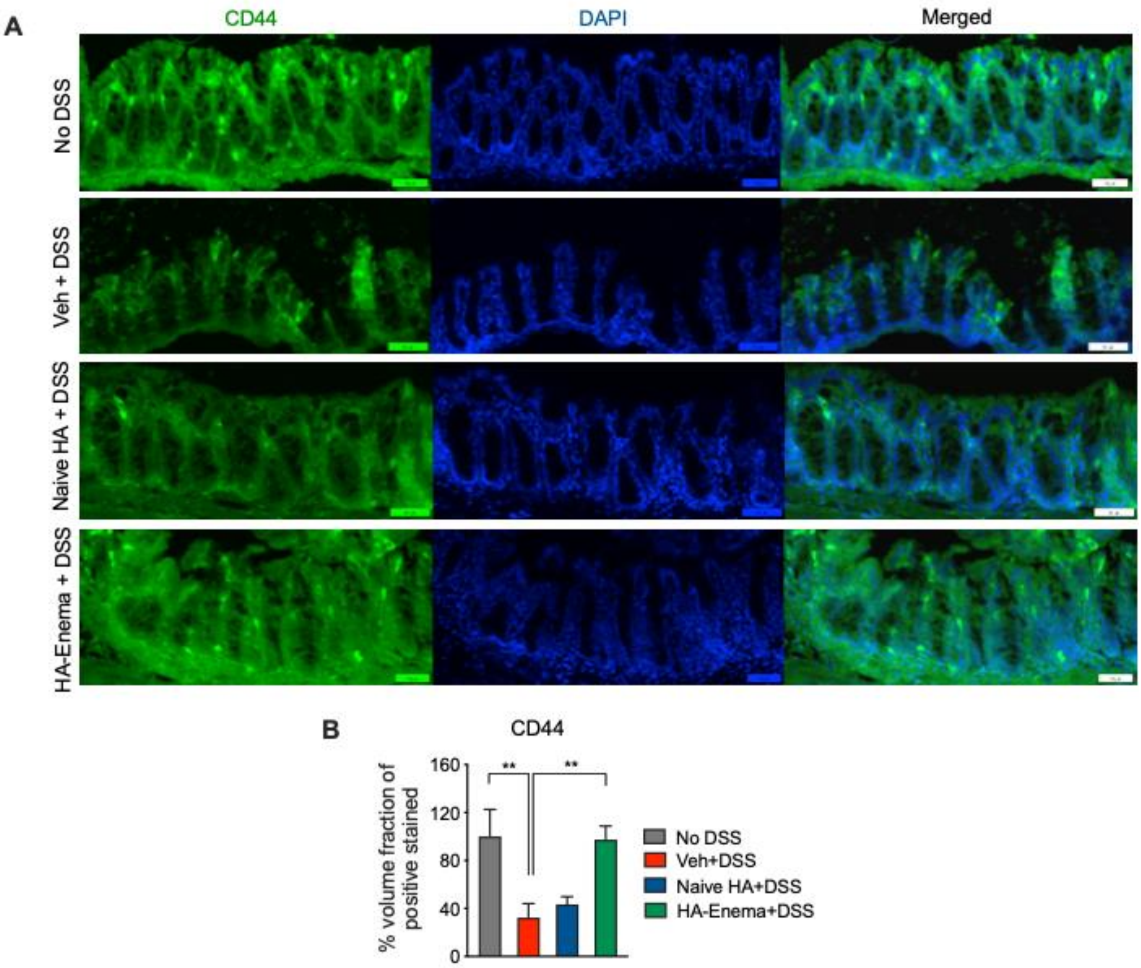

Figure S3. Proteomic changes (surface receptor) underlying intestinal epithelial layer following colitis induction and HA treatments. Representative immunofluorescence images showing presence of epithelial surface receptor CD44 at the colitis epithelial wall in HA-treated groups compared to vehicle (PBS) control group (A) and % volume fraction of the staining (B). Statistical analysis was performed using GraphPad Prism software by one-way ANOVA with Dunnett's multiple comparisons test, data were expressed as the mean ± SEM. $*p < 0.05$. Scale bar 50 µm.

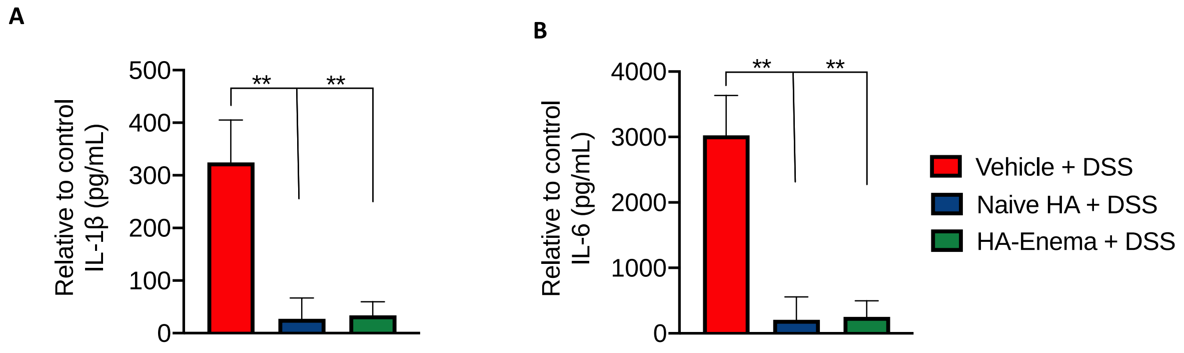

Figure S4. Proteomic changes (inflammatory markers) underlying intestinal epithelial layer following colitis induction and HA treatments. (A, B) Levels of IL-1β and IL-6 from the protein lysate of DSS colitis tissue after Naïve NA and HA-enema treatment. Statistical analysis was performed using GraphPad Prism software by one-way ANOVA with Dunnett's multiple comparisons test, data were expressed as the mean ± SEM. $^{**}p < 0.01$.
